# The Scanning CONfoCal Ophthalmoscopy foR DIAbetic eye screening (CONCORDIA) study paper 1

**DOI:** 10.1038/s41433-024-03360-2

**Published:** 2024-10-08

**Authors:** Peter H. Scanlon, Marta Gruszka-Goh, Ushna Javed, Anthony Vukic, Julie Hapeshi, Steve Chave, Paul Galsworthy, Scott Vallance, Stephen J. Aldington

**Affiliations:** 1 Gloucestershire Retinal Research Group (GRRG), Cheltenham General Hospital, Cheltenham, UK; 2 Nuffield Department of Clinical Neuroscience, University of Oxford, Cheltenham, UK; 3 University of Gloucestershire, Cheltenham, UK; 4 The Royal College of Ophthalmologists’ National Ophthalmology Audit, London, UK

**Keywords:** Public health, Medical imaging

## Abstract

**Objective:**

This project was to determine the performance of the Zeiss Clarus 700 (Clarus) and the Optos California (Optos) with staged mydriasis in a Diabetic Eye Screening Programme (DESP).

**Methods:**

Trial participants were recruited from people attending appointments in DESP or Virtual Eye clinics for delayed hospital appointments. Non-mydriatic photographs from the Clarus and Optos cameras were compared to 2-field 45 degrees mydriatic digital photography (the reference standard) and mydriatic photographs compared if the non-mydriatic photos were unassessable (staged mydriasis).

**Results:**

1573 patients were recruited. 76 individuals were withdrawn, leaving 1497 individuals (2993 eyes). For the Clarus and the Optos, the sensitivity for any retinopathy were 94.2% (95% CI: 92.9–95.3%) and 91.9% (95% CI: 90.5–93.2%) with specificities of 87.3% (95% CI: 85.4–89.0%) and 78.1% (95% CI: 75.7–80.3%) respectively. For referable DR the sensitivities for the Clarus and Optos were 86.0% (95% CI: 82.9–88.8%) and 77.6% (95% CI: 73.9–80.9%) with specificities of 92.8% (95% CI: 91.7–93.8%) and 95.4% (95% CI: 94.5–96.2%) respectively. The Clarus and Optos without mydriasis produced 100 (3.3%) and 152 (5.1%) unassessable eyes respectively, and after staged mydriasis 51 (1.7%) and 102 (3.4%) respectively with 52 (1.7%) reference standard images unassessable.

**Conclusions:**

This study reports the performance of the Clarus and the Optos using staged mydriasis in DR screening with wider fields detecting more referable retinopathy peripherally with some reduction in sensitivity centrally for macular lesions.

## Introduction

The English NHS Diabetic Eye Screening Programme [1] (NHS DESP) commenced in 2003 and, until 2023, offered annual screening using two 45-degree field digital imaging to each eye to all people with diabetes aged 12 years and older in England. In 2023, the screening interval was extended to 2-yearly for certain low risk groups who have had a negative screen on two consecutive occasions.

Scotland commenced [2] their screening programme in 2002 and offers staged mydriasis using one-45 degree field non-mydriatic imaging and only dilates those with an unassessable non-mydriatic image, which was reported [3] in 2020 to be 20% of those screened ranging from 8.9% of those aged 35–44 and 62% of those aged 85 years and older. The subject of this paper, is a clinical trial to determine the sensitivity and specificity of detection of any and referable retinopathy with staged mydriasis, using the Zeiss Clarus 700 camera (Carl Zeiss Ltd, Cambourne, UK) and the Optos California camera (Optos plc, Dunfermline, UK) within the central area covered by two 45-degree photographs taken in the English NHS DESP, which is the reference standard. The Zeiss Clarus 700 and the Optos California cameras are subsequently referred to as the Optos and the Clarus in this paper, except for one further clarification in the methods section. The Clarus camera is described as using Broad Line Fundus imaging and captures one field 90-degree image per eye with a combination of red, green and blue LED light and the Optos is described as a Scanning Confocal Ophthalmoscope (SCO) using red and green laser light to capture a field of approximately 135 degrees.

One of the earliest Optos devices (Optomap P200) was shown [4] to have a lower resolution centrally for the detection of microaneurysms (identifying 80.9% vs. 95%), despite capturing a much wider field. Recent studies have suggested that the resolution has improved [5, 6], that it performs well in non-mydriatic mode [7], but haven’t answered the question over whether the central resolution has improved sufficiently to detect screen positive diabetic maculopathy.

The Clarus was first introduced to the market in 2017 and so does not have the same historical literature and, although some studies have suggested that it is performing well in the detection of diabetic retinopathy [8–11], the numbers in these studies are relatively small, which was the reason to include the Clarus in this study.

This is the first paper from the CONCORDIA (Scanning CONfoCal Ophthalmoscopy foR DIAbetic eye screening) study.

The data were collected as part of an initial project in our programme of work entitled ‘AI Detection of Diabetic Retinopathy in Ultra-Wide-Field Retinal Images’ (AIDED), funded by Innovate UK. Anonymised data from AIDED was used for one of three work packages of the CONCORDIA study. The over-arching objective of the CONCORDIA programme of work is to determine if the cameras tested can be used in the non-mydriatic mode, and to determine if they are both effective and cost-effective to use in the NHS Diabetic Eye Screening Programme (DESP).

## Methods

**Fig. 1:**
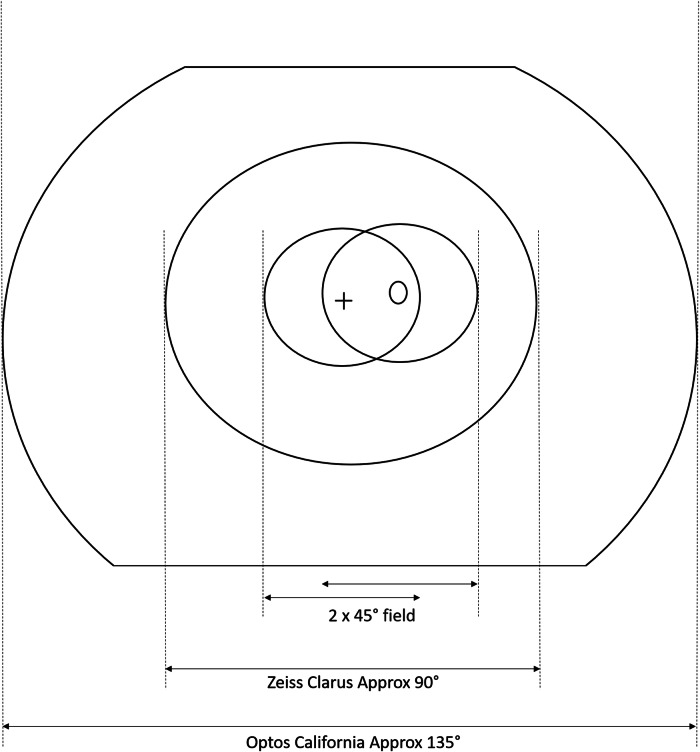
Diagram of areas of images from an Optos and Clarus superimposed on the two 45-degree fields.

We proposed a clinical trial to determine the sensitivity and specificity of the Zeiss Clarus 700 (Clarus) and the Optos California (Optos) using a staged mydriatic approach in a screening cohort setting to detect retinopathy lesions in the central area covered by the standard two 45-degree photographs as currently used in the NHS DESP. The staged mydriatic approach was chosen because there would be considerable advantages in using the devices if the majority of patients screened did not need their pupils dilated to take an assessable image and their use was found to be cost-effective. Both cameras cover a larger area of retina than that covered by the two 45-degree digital images as currently taken by the conventional cameras used in the English NHS DESP (Fig. 1).

The participants for the AIDED study, whose data were used in this analysis, were recruited during routine digital screening and digital surveillance clinics run by the Gloucestershire DESP. Study participants were also recruited from virtual eye clinics which had been set up to address the backlog of patients whose hospital ophthalmology follow up appointments were delayed because of the Covid-19 epidemic. These patients had a higher proportion of referable retinopathy than those in the routine digital screening clinics. Fundus imaging protocols within the virtual eye clinic were synonymous to DESP standards.

People in whom it was not possible to take retinal images, those with eye disease that might affect interpretation of diabetic retinopathy (DR) levels, and those under 16 years attending their first screening, were excluded from the study.

Reference standard images were taken after pupil dilation using non-mydriatic digital cameras used by the Gloucestershire DESP, which have been approved for use in the English NHS Diabetic Eye Screening Programme. The cameras used in the Gloucestershire DESP are the Canon CR2, Kowa AF, Topcon Triton and Topcon 2000.

When image quality was poor or pupil size was less than 3 mm, images were taken with the Clarus or the Optos, before and after dilation. Although Clarus marketing materials suggest 2.5 mm is their minimum capture range, the Optos manuals are more conservative at 3 mm. To avoid bias, 3 mm for all devices was chosen. The pupil size was estimated by the screener using an external ruler. The screeners in the English screening programme are not currently trained to assess whether an image is assessable at the time of capture. Hence it was decided to take a mydriatic image with the new cameras if the image was obviously unassessable or if the pupil size was less than 3 mm. Measurement of the pupil size was introduced after images had been taken on the first 343 eyes (11.5%) and hence took place on 2650 eyes (88.5%). The order of capture between the Clarus and the Optos cameras was randomised.

All images were graded by two graders with arbitration for differences of opinion by a third grader. The graders determined the retinopathy (R), maculopathy (M) and presence of photocoagulation scars (P) levels and also whether the images were assessable or not. Where images had been taken with and without mydriasis (e.g., in a patient with a pupil size less than 3 mm) and the non-mydriatic image was considered assessable by the graders, this was the image used for the comparisons of microaneurysm counts and for the sensitivity and specificity levels of any and referable DR.

All graders were either senior graders in the Gloucestershire DESP or graders in the English NHS DESP who are members of the Grading College, all of whom score highly in the monthly quality assurance Test and Training (TaT) sets that are taken by graders in the English NHS DESP. They were blinded to the randomisation of image capture and the type of clinic the images were captured from.

The senior graders in the Gloucestershire DESP have a track record of grading for a number of studies [12–14]. In the Emerald Study [14] these graders were trained on the interpretation of images from the Optos camera. Experienced graders were picked for the study who then undertook a 2-day face-to-face course followed by two additional half-day training sessions with a separate web-based teaching module being provided to consolidate knowledge. A test was then undertaken with a pass mark of 80%. With this background, the Gloucestershire graders received further training for this study from representatives of Clarus and Optos who delivered hands-on and virtual training sessions on image acquisition, lesion identification, and how to navigate the software. As the study incorporated new graders from the grading college, some of whom had also taken part in the Emerald study, these training elements were cascaded through documentation and virtual demonstrations by the grading coordinators. New graders were provided an initial set of images to grade, and their grading forms were quality checked. If these grading forms had any quality issues, these were communicated until proficiency was demonstrated.

The graders used both colour and red free tools to assess the images.

The Grading Form (Supplementary Table 1) is based on the grading form used by the NHS Diabetic Eye Screening Programme in England.

Supplementary Tables 2 and 3 compare the English NHS DESP grading form with the Early Treatment Diabetic Retinopathy Study [15] (ETDRS) and International Classification [16] for Diabetic Retinopathy and Maculopathy respectively.

To compare the same areas of the eyes, images from the Clarus and the Optos cameras were graded with a two 45-degree field overlay and all images graded using the NHS DESP grading criteria. In addition, microaneurysm counts were undertaken in the area of the 45-degree central macular field overlay. For the purposes of the analysis, the microaneurysm counts within 1 DD of the central fovea were used for comparison. They were recorded as 0,1,2,3,4,5+ and double graded and arbitrated. Ethics approval for the AIDED study was granted by the Health Research Authority with IRAS project ID 275896 on 14/02/20 and for some amendments on 20/07/20.

Ethics approval for the CONCORDIA study was granted by the Health Research Authority with IRAS project ID: 297725. REC reference: 21/SW/0064.

The clinical trial reference number was ISRCTN16254044.

The sample size was calculated using sample size formulae for calculating adequate sensitivity/specificity from Hajian-Tilaki [17].

Baseline characteristics of the population, by baseline DR severity and analysis cohorts, were summarised using descriptive statistics.

Sensitivity and specificity comparing the Clarus and the Optos cameras with the reference standard were calculated for any and referable levels of DR. The unassessable images for the reference standard images were excluded from the sensitivity, specificity, positive and negative predictive value analysis. All statistics were calculated with exact binomial confidence intervals.

## Results

1573 patients were recruited as trial participants and signed an informed consent. 76 individuals were withdrawn for the following reasons:

- imaging protocol deviation - 26 participants.
- patient withdrew consent and left before the imaging was complete – 12 participants.
- IT network error – 16 participants.
- recruitment / administrative error e.g., patient study ID not allocated – 22 participants.

The final sample consisted of 1497 individuals (2993 eyes) aged 15 to 94 years with diabetes mellitus that participated in the Diabetic Eye Screening Programme (routine, drop in and digital surveillance clinics) or attended Virtual Eye Clinics for delayed Hospital Eye Clinic appointments following the Covid-19 epidemic.

The trial cohort consisted of 1497 patients (2993 eyes). 906 (60.5%) of patients were male and 591 (39.5%) female.

Median age at the time of trial was 64.0 years with IQR (55.0–72.0) years and was one year higher for male patients. The most frequent (31.0%) trial participants were patients aged 65 to 74 years and the least frequent (1.1%) those aged between 15 and 24 years.

The majority (92.7%) of patients were of White Caucasian descent and proportions of individuals from other ethnic groups ranged between 0.9% and 3.1%, Table 1.

**Table 1:** Baseline characteristics of the trial patients.

	Gender		
	Female	Male	Total
*N*	591 (39.5%)	906 (60.5%)	1497 (100.0%)
Median Age (years)	63.0 (53.0–73.0)	64.0 (56.0–71.0)	64.0 (55.0–72.0)
Age Category (years)			
15–24	12 (2.0%)	5 (0.6%)	17 (1.1%)
25–34	27 (4.6%)	24 (2.6%)	51 (3.4%)
35–44	39 (6.6%)	49 (5.4%)	88 (5.9%)
45–54	79 (13.4%)	123 (13.6%)	202 (13.5%)
55–64	152 (25.7%)	268 (29.6%)	420 (28.1%)
65–74	168 (28.4%)	296 (32.7%)	464 (31.0%)
75–84	104 (17.6%)	122 (13.5%)	226 (15.1%)
85+	10 (1.7%)	19 (2.1%)	29 (1.9%)
Ethnicity			
White Caucasian	544 (92.0%)	843 (93.0%)	1387 (92.7%)
Asian or Asian British	20 (3.4%)	26 (2.9%)	46 (3.1%)
Black or Black British	15 (2.5%)	19 (2.1%)	34 (2.3%)
Mixed - Multiple Ethnic Groups	5 (0.8%)	8 (0.9%)	13 (0.9%)
Other	1 (0.2%)	2 (0.2%)	3 (0.2%)
Data Not Available	6 (1.0%)	8 (0.9%)	14 (0.9%)

From the 2993 eyes photographed pre-dilation using Clarus, 3.3% (100 eyes) with 95% CI (2.7–4.0%) were unassessable according to the grader. With staged mydriasis the number of unassessable eyes reduced to 1.7% (51 eyes) with 95% CI (1.3–2.2%).

From the 2993 eyes photographed pre-dilation using the Optos, 5.1% (152 eyes) with 95% CI (4.3–6.0%) were unassessable according to the grader. With staged mydriasis the number of unassessable eyes reduced to 3.4% (102 eyes) with 95% CI (2.8–4.2%).

The number of unassessable images from mydriatic digital photography equalled 1.7% (52 eyes) with 95% CI (1.3–2.3%).

Under the age of 55, only 7 eyes were unassessable in non-mydriatic Clarus imaging and 6 eyes using non-mydriatic Optos imaging. The age group of patients whose eyes were unassessable is shown in Table 2:

**Table 2:** Age group of patients whose eyes were unassessable.

Age category (years)		Clarus pre-dilation		Optos pre-dilation		Clarus Staged mydriasis		Optos Staged mydriasis		2-field mydriatic digital photography	
	Overall number of eyes	Number of eyes	Percentage of eyes	Number of eyes	Percentage of eyes	Number of eyes	Percentage of eyes	Number of eyes	Percentage of eyes	Number of eyes	Percentage of eyes
**12–24**	34	2	5.9%	1	2.9%	2	5.9%	1	2.9%	1	2.9%
**25–34**	102	0	0.0%	0	0.0%	0	0.0%	0	0.0%	0	0.0%
**35–44**	176	2	1.1%	1	0.6%	1	0.6%	1	0.6%	2	1.1%
**45–54**	404	3	0.7%	4	1.0%	3	0.7%	4	1.0%	3	0.7%
**55–64**	840	18	2.1%	26	3.1%	10	1.2%	17	2.0%	7	0.8%
**65–74**	927	45	4.9%	67	7.2%	20	2.2%	45	4.9%	19	2.0%
**75–84**	452	28	6.2%	47	10.4%	14	3.1%	30	6.6%	18	4.0%
**85** +	58	2	3.4%	6	10.3%	1	1.7%	4	6.9%	2	3.4%
**Total**	2993	100	3.3%	152	5.1%	51	1.7%	102	3.4%	52	1.7%

There were 25 eyes (0.8%) assessable using the non-mydriatic Clarus but unassessable on 2 × 45° mydriatic digital reference standard. There were 26 eyes (0.9%) that were assessable using the non-mydriatic Optos but unassessable on the 2 × 45° mydriatic digital reference standard.

For our first method of calculating sensitivity and specificity, we only analysed those eyes that were assessable on the two cameras being compared – the Clarus staged mydriatic images and the reference 2-field mydriatic digital images (2905 eyes assessable on both). Similarly, the Optos staged mydriatic images and the reference 2-field mydriatic digital images (2856 eyes assessable on both).

Grading results are shown in Tables 3 and 4.

**Table 3:** Grading results using staged mydriasis with the Zeiss Clarus.

Staged mydriasis Clarus		Reference standard														
		No DR	Non Referable DR		Referable DR											
		R0M0	R1M0 P0	R1M0 P1	R1M1 P0	R1M1 P1	R2M0 P0	R2M0 P1	R2M1 P0	R2M1 P1	R3M0 P0	R3M0 P1	R3M1 P0	R3M1 P1	R3MU P1*	Total (%)
**No DR**	**R0M0**	1,140	86	0	7	0	0	0	0	0	0	0	0	0	0	**1233 (42.4%)**
**Non-Referable DR**	**R1M0 P0**	143	770	3	61	0	9	1	1	0	0	0	1	0	0	**989 (34.0%)**
	**R1M0 P1**	2	5	9	0	1	0	0	0	0	0	0	0	0	0	**17 (0.6%)**
**Referable DR**	**R1M1 P0**	10	48	0	222	1	0	0	9	0	0	0	0	0	0	**290 (10.0%)**
	**R1M1 P1**	0	0	4	0	3	0	0	0	1	0	0	0	0	0	**8 (0.3%)**
	**R2M0 P0**	1	51	0	13	1	23	0	8	0	2	0	0	0	0	**99 (3.4%)**
	**R2M0 P1**	3	1	8	0	2	1	1	0	1	0	2	0	0	0	**19 (0.7%)**
	**R2M1 P0**	0	14	0	41	0	5	0	58	2	0	0	2	0	0	**122 (4.2%)**
	**R2M1 P1**	0	1	1	3	2	0	1	0	0	0	0	0	2	0	**10 (0.3%)**
	R2MU P1*	0	0	1	0	0	0	0	0	0	0	0	0	0	0	**1 (** < **0.1%)**
	**R3M0 P0**	0	6	0	2	0	5	1	3	0	5	0	0	0	0	**22 (0.8%)**
	**R3M0 P1**	6	3	5	0	0	1	2	0	1	0	12	0	3	0	**33 (1.1%)**
	**R3M1 P0**	0	0	0	4	0	2	0	15	0	0	0	7	0	0	**28 (1.0%)**
	**R3M1 P1**	1	0	2	2	2	0	1	0	3	1	4	3	10	2	**31 (1.1%)**
	R3MU P1*	0	0	1	0	0	0	0	0	0	0	2	0	0	0	**3 (0.1%)**
**Total (%)**		**1306 45.0%**	**985 33.9%**	**34 1.2%**	**355 12.2%**	**12 0.4%**	**46 1.6%**	**7 0.2%**	**94 3.2%**	**8 0.3%**	**8 0.3%**	**20 0.7%**	**13 0.5%**	**15 0.5%**	**20.1%**	**2905 (100.0%)**

Bold: the graders determined the retinopathy (R), maculopathy (M) and presence of photocoagulation scars (P) levels according to the grading form in Supplementary Table 1 that is used by the English NHS Diabetic Eye Screening Programme.

*Unassessable level of maculopathy.

**Table 4:** Grading results using staged mydriasis with the Optos California.

Staged mydriasis Optos		Reference standard														
		No DR	Non-Referable DR		Referable DR											
		R0M0	R1M0 P0	R1M0 P1	R1M1 P0	R1M1 P1	R2M0 P0	R2M0 P1	R2M1 P0	R2M1 P1	R3M0 P0	R3M0 P1	R3M1 P0	R3M1 P1	R3MU P1*	Total (%)
**No DR**	**R0M0**	993	120	2	5	0	0	0	0	0	0	0	0	0	0	**1120 (39.2%)**
**Non-Referable DR**	**R1M0P0**	263	766	0	90	0	14	0	10	0	1	0	0	0	0	**1145 (40.1%)**
	**R1M0P1**	6	6	20	0	2	0	1	0	2	0	2	0	1	0	**40 (1.4%)**
**Referable DR**	**R1M1P0**	2	53	0	228	1	0	0	14	0	0	0	0	0	0	**298 (10.4)**
	**R1M1P1**	0	0	3	1	4	0	0	0	0	0	0	0	0	0	**8 (0.3%)**
	R1MUP0*	0	0	0	1	0	0	0	0	0	0	0	0	0	0	**1 (** < **0.1%)**
	**R2M0P0**	1	19	0	12	0	17	1	9	1	0	0	0	0	0	**60 (2.1%)**
	**R2M0P1**	1	1	8	0	1	1	3	1	1	0	0	0	0	0	**17 (0.6%)**
	**R2M1P0**	0	4	0	11	0	11	0	54	0	0	1	1	0	0	**82 (2.9%)**
	**R2M1P1**	0	0	0	2	2	1	0	0	2	0	1	0	2	0	**10 (0.4%)**
	R2MUP0*	0	0	0	1	0	0	0	1	0	0	0	0	0	0	**2 (0.1%)**
	R2MUP1*	0	0	0	0	0	0	1	0	0	0	0	0	0	0	**1 (** < **0.1%)**
	**R3M0P0**	0	3	0	1	0	1	0	1	0	4	0	0	0	0	**10 (0.4%)**
	**R3M0P1**	3	2	1	0	0	0	1	0	0	2	13	0	6	0	**28 (1.0%)**
	**R3M1P0**	1	0	0	0	0	1	0	3	0	0	0	8	0	0	**13 (0.5%)**
	**R3M1P1**	0	0	1	1	2	0	0	1	2	0	3	3	6	1	**20 (0.7%)**
	R3MUP1*	2	0	0	0	0	0	0	0	0	0	0	0	0	0	**2 (0.1%)**
**Total (%)**		**1272 44.5%**	**975 34.1%**	**35 1.2%**	**353 12.4%**	**12 0.4%**	**46 1.6%**	**7 0.3%**	**94 3.3%**	**8 0.3%**	**7 0.3%**	**20 0.7%**	**12 0.4%**	**15 0.5%**	**1** < **0.1%**	**2856 100%**

Bold: the graders determined the retinopathy (R), maculopathy (M) and presence of photocoagulation scars (P) levels according to the grading form in Supplementary Table 1 that is used by the English NHS Diabetic Eye Screening Programme.

*Unassessable level of maculopathy.

57.6% of images taken with the Clarus and 55.0% taken with 2-field mydriatic digital photography have been graded as those with any diabetic retinopathy. For the Clarus, the sensitivity and specificity for any retinopathy were 94.2% with 95% CI (92.9–95.3%) and 87.3% with 95% CI (85.4–89.0%) respectively. Positive predictive value was 90.1% with 95% CI (88.5–91.5%) and negative predictive value 92.5% with 95% CI (90.8–93.9%). There was near perfect inter-grader agreement in detecting any DR between graders assessing Clarus images (Cohen’s Kappa = 0.839).

60.8% of images taken with Optos and 55.5% taken with 2-field mydriatic digital photography have been graded as those with any diabetic retinopathy. For the Optos, the sensitivity and specificity for any retinopathy were 91.9% with 95% CI (90.5–93.2%) and 78.1% with 95% CI (75.7–80.3%) respectively. Positive predictive value for any retinopathy was 83.9% with 95% CI (82.1–85.6%) and negative predictive value 88.6% with 95% CI (86.6–90.4%). There was substantial inter-grader agreement in detecting any DR between graders assessing Optos images (Cohen’s Kappa = 0.632).

22.9% of images taken with the Clarus and 20.0% taken with 2-field mydriatic digital photography have been graded as those with referable diabetic retinopathy. For the Clarus, the sensitivity and specificity for referable retinopathy were 86.0% with 95% CI (82.9–88.8%) and 92.8% with 95% CI (91.7–93.8%) respectively. Positive predictive value was 74.9% with 95% CI (71.5–78.2%) and negative predictive value 96.4% with 95% CI (95.5–97.1%). There was substantial inter-grader agreement in detecting referable DR between graders assessing Clarus images (Cohen’s Kappa = 0.775).

The difference in sensitivity of referable DR was mostly due to grading of maculopathy, where the differences were caused by:

- grader interpretation - 13
- different appearance of lesion - 20
- lesion not observed on the Clarus - 15
- lesion masked by artefact - 8
- whether fine exudates were seen within 1 disc diameter (1DD) of the centre of the fovea - 5.
  For our second method of calculating sensitivity and specificity, counting the images that were unassessable on the Clarus but were assessable on the 2-field digital imaging as test positive the corresponding sensitivity and specificity for any DR was 94.2% (95% CI: 93.0–95.3%) and 86.1% (95% CI: 84.1–87.9%), and the sensitivity and specificity for referable DR was 96.1% (95% CI: 83.0–88.8%) and 91.5% (95% CI: 90.3–92.6%), respectively.
  There were 36 images that were considered assessable on the Clarus but were unassessable on the reference standard 2-field digital images.
  19.3% of images taken with the Optos and 20.1% taken with 2-field mydriatic digital photography have been graded as those with referable diabetic retinopathy. For the Optos, using our first method of calculation described above, the sensitivity and specificity for referable retinopathy were 77.6% with 95% CI (73.9–80.9%) and 95.4% with 95% CI (94.5–96.2%) respectively. Positive predictive value was 80.9% with 95% CI (77.4–84.1%) and negative predictive value 94.4% with 95% CI (93.4–95.3%). There was substantial inter-grader agreement in detecting referable DR between graders assessing Optos images (Cohen’s Kappa = 0.617).
  The difference in sensitivity of referable DR was mostly due to grading of maculopathy, where the differences were caused by:
- grader interpretation - 20
- different appearance of lesion - 21
- lesion not observed on the Optos - 29
- lesion masked by artefact - 16
- whether fine exudates were seen within 1 disc diameter of the centre of the fovea - 4.

Using our second method of calculation, counting the images that were unassessable on the Optos but were assessable on the 2-field digital imaging as test positive the corresponding sensitivity and specificity for any DR was 92.1% (95% CI: 90.7–93.4%) and 75.0% (95% CI: 72.6–77.3%), and the sensitivity and specificity for referable DR was 77.9% (95% CI: 74.3–81.2%) and 92.3% (95% CI: 91.1–93.3%), respectively.

There were 85 images that were considered assessable on the Optos but were unassessable on the reference standard 2-field digital images.

The microaneurysm (MA) count was defined as non-inferior, when the number of microaneurysms detected within 1DD of the centre of the fovea on images using the Clarus or the Optos was equal to or higher than on the images taken using two 45-degree field digital cameras.

For staged-mydriasis images taken with the Clarus, in 91.2% of cases, the images were non-inferior in a detection of MA counts within 1DD of the centre of the fovea to the mydriatic digital photography used as a diabetic screening standard.

Out of 2993 images taken following staged-dilation process using the Clarus, the median number of MA counts was 0.0 with IQR (0.0–1.0) and mean MA count was 0.8 with SD (1.6).

The median number of MA counts for mydriatic digital photography equalled 0.0 with IQR (0.0–0.0) and mean MA count was 0.6 with SD (1.3).

For staged-mydriasis images taken with the Optos, in 89.7% of cases, the images were non-inferior in a detection of MA counts within 1DD of the centre of the fovea to the mydriatic digital photography used as a diabetic screening standard.

Out of 2993 images taken following staged-dilation process using the Optos, the median number of MA counts was 0.0 with IQR (0.0–1.0) and mean MA count was 0.8 with SD (1.5).

The median number of MA counts for mydriatic digital photography equalled 0.0 with IQR (0.0–0.0) and mean MA count was 0.6 with SD (1.3).

Additional diabetic retinopathy lesions outside the 2 × 45-degree were detected by the Clarus for 129 (4.3%) eyes:

- 82 (2.7% of eyes) additional cases of non-referable diabetic retinopathy.
- 23 (0.8% of eyes) additional cases of referable diabetic retinopathy.
- 24 (0.8% of eyes) additional cases of higher level of referable retinopathy.
  Additional diabetic retinopathy lesions were detected outside the 2 × 45-degree by the Optos for 347 (11.5%) eyes:
- 242 (8.1% of eyes) additional cases of non-referable diabetic retinopathy.
- 57 (1.9% of eyes) additional cases of referable diabetic retinopathy.
- 38 (1.3% of eyes) additional cases of higher level of referable retinopathy.
- 5 (0.2% of eyes) cases of eyes moving from no retinopathy into referable retinopathy category.
- 3 (0.1% of eyes) cases of unassessable eyes moving into referable retinopathy category,

## Discussion

In order for new camera technology to be accepted for use in the English NHS Diabetic Eye Screening Programme it will be required to:

1. have a high sensitivity for the detection of any retinopathy and referable retinopathy compared to the current methodology of 2-field digital imaging.
2. have a high specificity for the detection of any retinopathy and referable retinopathy.
3. be shown to be cost-effective for use in the screening programme.
  There would also be advantages if the technology:
4. could be used without eye drops in the majority using staged mydriasis.
5. detected other significant lesions outside the current area captured by the 2-field digital imaging.

Both the Clarus and the Optos had high sensitivities of over 90% for the detection of any diabetic retinopathy. However, they both had reduced sensitivities for detection of referable retinopathy at 86% for the Clarus and 77.8% for the Optos. The reduction in sensitivities of referable DR for the Clarus and the Optos were mostly due to grading of maculopathy where the experienced grading team found some novel presentations of acquisition artefacts and colour differences in DR features. The colour differences did not affect red lesions (e.g., microaneurysms, haemorrhages, IRMA and new vessels) but made the interpretation of drusen and hard exudate more difficult for graders, which would have training implications if these cameras were introduced into the English NHS DESP. This would explain the differences in these results and the study [6] conducted by the DRCR.net. which did not include any grading of maculopathy and excluded patients with centre involving diabetic macular oedema.

The specificities for detection of any retinopathy were 87.3% for the Clarus and 78.1% for the Optos. For referable retinopathy, the specificities for the Clarus and the Optos were 92.8% and 95.4% respectively. The specificity for any retinopathy for the Optos was reduced to 78.1% because of the higher number of false positives when grading Optos images.

A cost-effectiveness analysis is being undertaken as a separate workpackage of this CONCORDIA study.

Both cameras performed extremely well using staged mydriasis where only 1.7% of eyes captured with the Clarus and 3.4% of eyes captured with the Optos required dilation in this predominantly White Caucasian population.

A preliminary analysis shows that the Clarus and the Optos detected an additional 1.6% and 3.2% of eyes respectively with referable retinopathy. Further analyses will be undertaken which will be the subject of a separate paper.

The overall results of this study were promising for both cameras, especially when one considers how well they both performed using staged mydriasis. However, as 92.7% of the participants in this study came from a White Caucasian background, the results need to be tested in other ethnic groups.

## Summary

### What was known before:

- These cameras are currently not approved for use within a UK screening programme because there have been concerns over their central resolution.

### What this study adds:

- This is the first study that compares the new widefield devices against the standard screening photography in the central 2 × 45 degree fields that are used in the NHS Diabetic Eye Screening Programme.

## Supplementary information

Supplementary Table 1

Supplementary Table 2

Supplementary Table 3

## Data Availability

All data supporting the findings of this study are available within the paper and its Supplementary Information.
