# Supplementary material for: The Scanning CONfoCal Ophthalmoscopy foR DIAbetic eye screening (CONCORDIA) study paper 1
Source: Eye (Lond). 2024 Oct 8;38(18):3539–46. doi: 10.1038/s41433-024-03360-2 (PMC11621412; doi:10.1038/s41433-024-03360-2)
Supplement: Supplementary file 1 — Supplementary Table 1 [file 41433_2024_3360_MOESM1_ESM.docx]

**Supplementary Table 1** - Grading form

| **FEATURE BASED GRADING** | **Right eye** | Left eye |
| --- | --- | --- |
| **ASSESSABILITY** | ASSESSABLE / UNASSESSABLE | ASSESSABLE / UNASSESSABLE |
|  |  |  |
| **R0 – NO DIABETIC RETINOPATHY** | YES / NO | YES / NO |
|  |  |  |
| **R1 – BACKGROUND DIABETIC RETINOPATHY** | |  |
| MICROANEURYSMS | YES / NO | YES / NO |
| RETINAL HAEMORRHAGE | YES / NO | YES / NO |
| VENOUS LOOP | YES / NO | YES / NO |
| EXUDATE | YES / NO | YES / NO |
| COTTON WOOL SPOT | YES / NO | YES / NO |
| **MICROANEURYSM COUNT** |  |  |
| Within 1DD of fovea | 0, 1, 2, 3, 4, 5+ | 0, 1, 2, 3, 4, 5+ |
|  |  |  |
| **R2 – PRE-PROLIFERATIVE DIABETIC RETINOPATHY** | |  |
| VENOUS BEADING | YES / NO | YES / NO |
| VENOUS REDUPLICATION | YES / NO | YES / NO |
| IRMA (INTRARETINAL MICROVASCULAR ABNORMALITY) | YES / NO | YES / NO |
| MBH (MULTIPLE BLOT HAEMORRHAGES) | YES / NO | YES / NO |
|  |  |  |
| **R3 – PROLIFERATIVE DIABETIC RETINOPATHY** | |  |
| NVE (NEW VESSELS ELSEWHERE) | YES / NO | YES / NO |
| NVD (NEW VESSELS AT DISC) | YES / NO | YES / NO |
| PRE-RETINAL HAEMORRHAGE | YES / NO | YES / NO |
| PRE-RETINAL FIBROSIS | YES / NO | YES / NO |
| VITREOUS HAEMORRHAGE | YES / NO | YES / NO |
| RETINAL DETACHMENT WITH DR | YES / NO | YES / NO |
|  |  |  |
| **M0 – NO DIABETIC MACULOPATHY** | YES / NO | YES / NO |
|  |  |  |
| **M1 – DIABETIC MACULOPATHY** |  |  |
| EXUDATE WITHIN 1DD OF CENTRE OF FOVEA | YES / NO | YES / NO |
| GROUP OF EXUDATES WITHIN THE MACULA,  GREATER THAN OR EQUAL TO HALF THE DISC AREA | YES / NO | YES / NO |
| ANY MICROANEURYSM OR HAEMORRHAGE  WITHIN 1DD OF CENTRE OF FOVEA WITH A BEST VA WORSE THAN 0.3 | YES / NO | YES / NO |
|  |  |  |
| **PHOTOCOAGULATION** |  |  |
| P0 – NO LASER SCARS | YES / NO | YES / NO |
| P1 – FOCAL LASER SCARS | YES / NO | YES / NO |
| P1 – PAN-RETINAL LASER SCARS | YES / NO | YES / NO |
|  |  |  |
| **Other Conditions (Non-DR)** |  |  |
| Suspicious Non-DR Lesion | YES / NO | YES / NO |
|  |  |  |
| **FINAL GRADES** | R#M#P# | R#M#P# |
