# Supplementary material for: The Scanning CONfoCal Ophthalmoscopy foR DIAbetic eye screening (CONCORDIA) study paper 1
Source: Eye (Lond). 2024 Oct 8;38(18):3539–46. doi: 10.1038/s41433-024-03360-2 (PMC11621412; doi:10.1038/s41433-024-03360-2)
Supplement: Supplementary file 2 — Supplementary Table 2 [file 41433_2024_3360_MOESM2_ESM.docx]

**Supplementary Table 2**

Comparison of Diabetic Retinopathy Classifications for Retinopathy ‘R’ levels and Risk of Progression

| **ETDRS final Retinopathy Severity Scale** | **ETDRS**  **Grade** | **Risk of progression to Proliferative DR (PDR) in 1 year** | **Lesions** | **‘International’ Clinical Classification of Diabetic Retinopathy Severity** | **English Screening Programme levels** |
| --- | --- | --- | --- | --- | --- |
| No apparent retinopathy | 10  14, 15 |  | DR absent  DR questionable |  | **R0**  Currently screen  Annually |
| Mild NPDR | 20 |  | Micro aneurysms only | Ma’s only | **R1**  Screen annually  **Background** microaneurysm(s)  Retinal haemorrhage(s) ± any exudate |
|  | 35 | Level 30 = 6.2% | One or more of the following:  Venous loops > definite in 1 field  SE, IRMA, or VB questionable  Retinal haemorrhages present  HE > definite in 1 field  SE > definite in 1 field | More than just micro aneurysms but less severe than Severe NPDR |  |
| Moderate NPDR | 43 | Level 41 = 11.3% | H/Ma moderate in 4-5 fields or severe in 1 field  or  IRMA definite in 1-3 fields |  | **R2**  Refer to ophthalmologist  **Pre-proliferative**  venous beading  venous reduplication  intraretinal microvascular abnormality (IRMA)  multiple deep, round or blot haemorrhages |
| Moderately severe NPDR | 47 | Level 45 = 20.7% | Both level 43 characteristics - H/Ma moderate in 4-5 fields or severe in 1 field and IRMA definite in 1-3 fields  **and / or** any one of the following:  IRMA in 4-5 fields  VB definite in 1 field | Severe NPDR  Any of the following:  a) Extensive intraretinal haem (>20) in 4 quadrants  b) Definite venous beading in 2+ quadrants  c) Prominent IRMA in 1+ quadrant  And no signs of PDR |  |
| Severe NPDR | 53 | Level 51 = 44.2%  Level 55 = 54.8% | One or more of the following:  > 2 of the 3 level 47 characteristics  H/Ma severe in 4-5 fields  IRMA > moderate in 1 field  VB > definite in 2-3 fields |  |  |
| Mild PDR | 61 |  | FPD or FPE present with NVD absent or NVE = definite | Neovascularisation  Vitreous / preretinal haemorrhage | **R3**  **Proliferative**  new vessels on disc (NVD)  new vessels elsewhere (NVE)  pre-retinal or vitreous haemorrhage  pre-retinal fibrosis ± tractional retinal detachment |
| Moderate PDR | 65 |  | 1. NVE > moderate in 1 field or definite NVD with VH and PRH absent or questionable   or   1. VH or PRH definite and NVE < moderate in 1 field and NVD absent |  |  |
| High risk PDR | 71 |  | Any of the following:   1. VH or PRH > moderate in 1 field 2. NVE > moderate in 1 field and VH or PRH definite in 1 field 3. NVD = 2 and VH or PRH definite in 1 field 4. NVD > moderate |  |  |
| High risk PDR | 75 |  | NVD > moderate and definite VH or PRH |  |  |
