# Supplementary material for: The Scanning CONfoCal Ophthalmoscopy foR DIAbetic eye screening (CONCORDIA) study paper 1
Source: Eye (Lond). 2024 Oct 8;38(18):3539–46. doi: 10.1038/s41433-024-03360-2 (PMC11621412; doi:10.1038/s41433-024-03360-2)
Supplement: Supplementary file 3 — Supplementary Table 3 [file 41433_2024_3360_MOESM3_ESM.docx]

**Supplementary Table 3 -** Comparison of Classifications for Maculopathy ‘M’ levels

| Early Treatment Diabetic Retinopathy Study | International classification | English Classification |
| --- | --- | --- |
|  | Diabetic Macular Oedema Present as defined by some retinal thickening or hard exudates in the posterior pole and subclassified into:  Mild Diabetic Macular Oedema:  Some retinal thickening or hard exudates in the posterior pole but distant from the macula | Circinate or group of exudates within the macula  (The macula is defined as that part of the retina which lies within a circle centred on the centre of the fovea whose radius is the distance between the centre of the fovea and the temporal margin of the disc) |
|  |  | Any microaneurysm or haemorrhage within 1DD of the centre of the fovea only if associated with a best VA of ≤ 6/12 (if no stereo) |
| Clinically significant macular oedema as defined by: | Moderate diabetic macular oedema:  Retinal thickening or hard exudates approaching the centre of the macula but not involving the centre | Exudate within 1 disc diameter (DD) of the centre of the fovea |
| A zone or zones of retinal thickening one disc area or larger, any part of which is within one disc diameter of the centre of the macula. |  | Retinal thickening within 1DD of the centre of the fovea (if stereo available) |
| Retinal thickening at or within 500 microns of the centre of the macula | Severe diabetic macular oedema:  Retinal thickening or hard exudates involving the centre of the macula |  |
| Hard exudates at or within 500 microns of the centre of the macula, if associated with thickening of the adjacent retina (not residual hard exudates remaining after disappearance of retinal thickening) |  |  |
